# Ikoma Lyssavirus, Highly Divergent Novel Lyssavirus in an African Civet^1^

**DOI:** 10.3201/eid1804.111553

**Published:** 2012-04

**Authors:** Denise A. Marston, Daniel L. Horton, Chanasa Ngeleja, Katie Hampson, Lorraine M. McElhinney, Ashley C. Banyard, Daniel Haydon, Sarah Cleaveland, Charles E. Rupprecht, Machunde Bigambo, Anthony R. Fooks, Tiziana Lembo

**Affiliations:** Animal Health and Veterinary Laboratories Agency, Addlestone, UK (D.A. Marston, D.L. Horton, L.M. McElhinney, A.C. Banyard, A.R. Fooks);; Central Veterinary Laboratory, Dar es Salaam, Tanzania (C. Ngeleja);; University of Glasgow, Glasgow, Scotland, UK (K. Hampson, D. Haydon, S. Cleaveland, T. Lembo);; National Consortium for Zoonosis Research, Neston, UK (L.M. McElhinney, A. R. Fooks);; Centers for Disease Control and Prevention, Atlanta, Georgia, USA (C.E. Rupprecht);; Lincoln Park Zoo Tanzania Program, Arusha, Tanzania (M. Bigambo)

**Keywords:** Tanzania, African civet, rabies virus, West Caucasian bat virus, rabies virus, viruses, Lyssavirus, lyssaviruses, Ikoma lyssavirus, novel rabies virus, novel lyssavirus

## Abstract

Evidence in support of a novel lyssavirus was obtained from brain samples of an African civet in Tanzania. Results of phylogenetic analysis of nucleoprotein gene sequences from representative *Lyssavirus* species and this novel lyssavirus provided strong empirical evidence that this is a new lyssavirus species, designated Ikoma lyssavirus.

Eleven *Lyssavirus* species have been classified: *Rabies virus* (RABV), *Lagos bat virus* (LBV), *Mokola virus* (MOKV), *Duvenhage virus* (DUVV), *European bat lyssavirus* types -1 and -2, *Australian bat lyssavirus*, *Aravan virus*, *Khujand virus*, *Irkut virus*, and *West Caucasian bat virus* (WCBV) (*1*). All these viruses except MOKV have been detected in bats. Two newly identified lyssaviruses, Shimoni bat virus (SHIBV) (*2*) and Bokeloh bat lyssavirus (*3*), both detected in bats, have not yet been classified. The presence of numerous lyssaviruses in bat species has led to increasing research efforts toward lyssavirus discovery in bat populations globally. However, lyssavirus surveillance in terrestrial mammals remains limited across most of Africa.

Of the 13 lyssaviruses, 5 circulate in Africa (RABV, LBV, MOKV, DUVV, and SHIBV). LBV, MOKV, DUVV, and SHIBV are detected exclusively in Africa, whereas RABV is detected worldwide. The predominant RABV variants circulating in Africa are the mongoose and canine biotypes. In South Africa, canine RABV is considered to have been introduced in the eastern Cape Province after importation of an infected dog from England in 1892 and subsequently spread, infecting domestic and wild carnivores (*4*). Separate introductions of canine RABV (particularly in northern Africa) have been suggested (*5*). In addition, molecular clock analysis indicates that mongoose RABV was present in southern Africa ≈200 years before the introduction of canine RABV (*6*).

In Tanzania, canine RABV is endemic and widespread throughout the country. In the Serengeti ecosystem, detailed studies have shown a single variant of canine RABV circulating in multiple host species (*7*). However, annual mass rabies vaccination campaigns have been conducted for dogs in villages surrounding Serengeti National Park since 2003, and rabies has not been detected in the park since 2000 (*8*). Enhanced laboratory-based surveillance in support of this canine rabies elimination program has been running concurrently in the region.

## The Study

**Figure 1:**
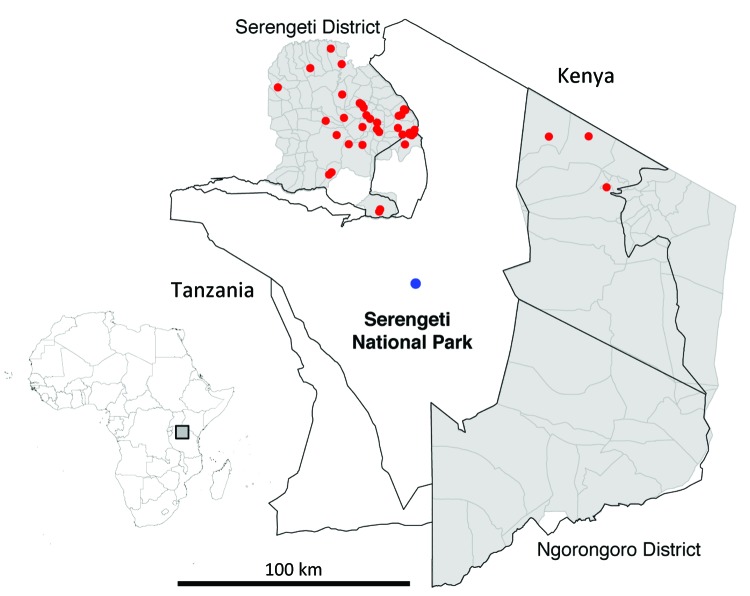
Serengeti National Park and surrounding districts (Serengeti and Ngorongoro). Blue dot indicates location of Ikoma lyssavirus–infected African civet within Ikoma Ward in northwest Tanzania. Red dots indicate cases of rabies confirmed during 2003–2011. Top left, map of Africa indicating study area in Tanzania (gray box).

On May 11, 2009, an African civet (*Civettictis civetta*) displaying clinical signs consistent with rabies was killed by rangers in Ikoma Ward within Serengeti National Park (Figure 1). Rangers were contacted because the civet had bitten a child on the right leg in an unprovoked attack. The wound was washed with soap and water, and the child received postexposure rabies vaccination but no rabies immunoglobulin. Brain samples from the civet were tested multiple times (as part of a training course) at the Central Veterinary Laboratory in Tanzania. Results of the fluorescent antibody test and a direct rapid immunohistochemistry test were positive for lyssavirus-specific antigen. When testing was complete, the samples were sent to the Animal Health and Veterinary Laboratories Agency (AHVLA, Weybridge, UK) for additional confirmation of results and molecular analysis.

**Figure 2:**
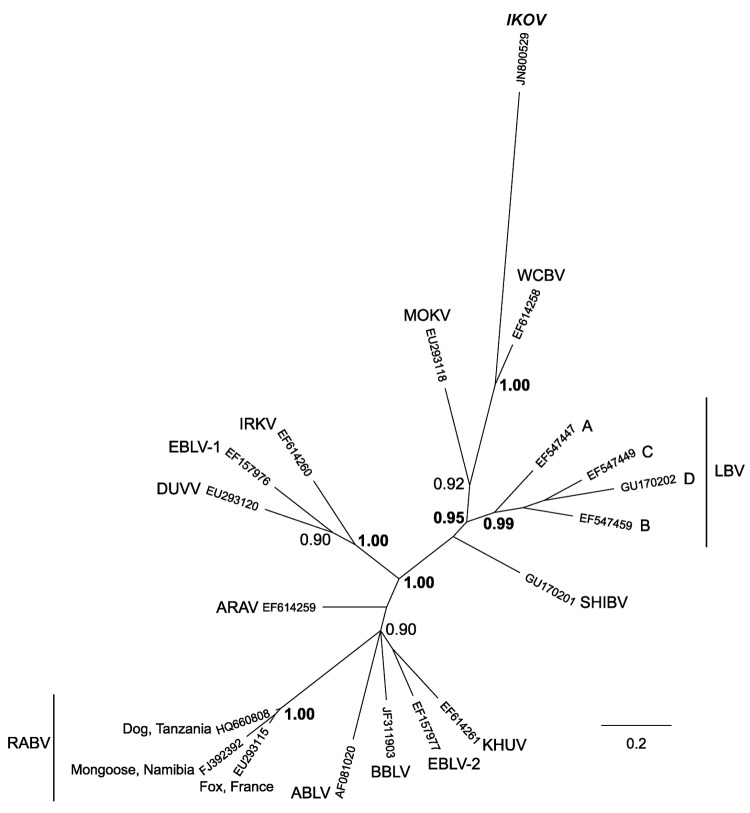
Phylogenetic relationships between all currently identified lyssaviruses compared with Ikoma lyssavirus (IKOV; shown in ***boldface italics***), as determined on the basis of partial nucleoprotein gene sequences (405 bp). Relationships are presented as an unrooted phylogram based on Bayesian Markov chain Monte Carlo (MCMC) analysis. Sequences were aligned by using ClustalX (version 2.0.10; www.clustal.org/clustal2). The MCMC analysis was performed in MrBayes (version 3.1.2; http://mrbayes.sourceforge.net/), by using the general-time reversible model with a proportion of invariable sites and a gamma-shaped distribution of rates across sites, treating values for model parameters as unknown variables with uniform priors to be estimated in each analysis. Analyses were conducted with 2 independent runs initiated with random starting trees without constraints. Four simultaneous MCMC chains (3 heated and 1 cold, according to the default settings) were run for 10^7^ generations; trees were sampled every 100 generations. The first 25,000 trees were discarded as the burn-in phase; the remaining trees were used to estimate consensus phylograms and Bayesian posterior probabilities. MrBayes outputs were examined by using Tracer 1.5 (http://beast.bio.ed.ac.uk/Tracer). The phylogenetic tree was visualized by using FigTree (version 1.3.1; http://beast.bio.ed.ac.uk/FigTree). Posterior probability values represent the degree of support for each node on the tree: only values >0.90 are shown; values >0.95 are shown in **boldface**. Scale bar indicates branch length, expressed as the expected number of substitutions per site. ARAV, Aravan virus; ABLV, Australian bat lyssavirus; BBLV, Bokeloh bat lyssavirus; DUVV, Duvenhage virus; EBLV-1 and EBLV-2, European bat lyssavirus type 1 and 2; IRKV, Irkut virus; KHUV, Khujand virus; LBV, Lagos bat virus (lineages A, B, C and D); MOKV, Mokola virus; RABV, rabies virus; SHIBV, Shimoni bat virus; WCBV, West Caucasian bat virus.

RNA was extracted by using TRIzol Reagent (Invitrogen, Paisley, UK), and a pan-lyssavirus reverse transcription PCR yielded a specific 606-bp amplicon (*9*). The amplicon (GenBank accession no. JN800509) was sequenced by using standard primers and protocols (*10*). Bayesian reconstructions were used for phylogenetic analysis of the nucleoprotein gene region (405 bp) and included representatives of all species from the lyssavirus genus; results showed that the sequence was unique and most closely related to WCBV (Figure 2). A canine RABV biotype from Tanzania and a mongoose RABV biotype from southern Africa were included in the dataset. Nucleotide comparisons indicated similar divergence from all lyssavirus species (minimum identity 62.2% *Australian bat lyssavirus*, maximum identity 68.6% WCBV), including 12 canine RABV sequences from domestic and wild animals in the Serengeti ecosystem (64.1%–65.1% identity). The posterior probabilities indicated that the IKOV and WCBV grouping was strongly supported, despite low sequence identity. Further phylogenetic analysis of representatives from other rhabdoviruses demonstrated that IKOV is a member of the *Lyssavirus* genus (41.6%–50.9% identity to representative rhabdovirus sequences that are available for this region of the genome) (data not shown).

## Conclusions

We describe evidence, based on genomic sequences obtained from the brain sample of an African civet with clinical signs consistent with rabies, for the existence of a novel lyssavirus designated IKOV (Ikoma lyssavirus). The sample was frozen and thawed several times before being sent to AHVLA, which had a detrimental effect on the sample quality and resulted in viral RNA degradation and loss in viral viability. The results of confirmatory FATs performed at AHVLA were inconclusive, and attempts to isolate virus by using the rabies tissue culture inoculation test and the mouse inoculation test were unsuccessful. Despite the lack of isolated virus, the pan-lyssavirus sensitivity and specificity of the fluorescent antibody test (the test prescribed by the World Organisation for Animal Health as the standard for rabies testing) and direct rapid immunohistochemistry test support the assertion that a novel lyssavirus exists in the region. In addition, despite the poor quality of the sample, molecular techniques identified a lyssavirus-specific amplicon that was confirmed to be unique by phylogenetic analysis and to be highly divergent from known circulating RABV strains. A real-time PCR also detected this unique lyssavirus sequence, confirming that both molecular tests are pan-lyssavirus specific and are sufficient for the detection of highly divergent novel lyssaviruses (*11*).

The child who was bitten by the African civet received appropriate wound care and postexposure rabies vaccination. At the time of this report, the child remained well. We cannot, however, draw any conclusions as to whether the African civet was shedding virus when it bit the child or whether postexposure vaccinations are effective against IKOV.

This case of rabies in an African civet in the center of Serengeti National Park was highly unexpected. Since 2000, the park had been free of rabies and no cases had been detected within a 30-km radius. This case of rabies in wildlife implied a major breach in the vaccination program. However, subsequent molecular characterization demonstrated that this case of rabies had not been caused by a RABV from a canine source. Thus, a breach had not occurred; instead, a novel lyssavirus with an unknown reservoir had caused the infection. Previously published data on lyssavirus infection in African civets (n = 6) was restricted to the RABV mongoose lineage (*12*). Although African civets can be infected with RABV and IKOV, infrequent detection of lyssaviruses in this species suggests that they are more likely to be incidental hosts. The nocturnal, opportunistic foraging behaviors of African civets imply that contact with bats is possible, particularly at roosts where interactions with a grounded rabid bat are more likely to occur. In the absence of virus isolates, the origin of IKOV is difficult to determine. Surveillance for rabies in bats and other mammals in Tanzania and typing of all lyssavirus-positive samples is necessary to determine the distribution and prevalence of IKOV.

The detection of WCBV cross-reacting neutralizing antibodies in gregarious *Miniopterus* spp. bats in neighboring Kenya could be informative, given the strong posterior probability values on the grouping of IKOV and WCBV in the Bayesian analysis (Figure 2) (*13*). Additional genomic and evolutionary analysis is underway to support IKOV as a new *Lyssavirus* species, potentially grouping with WCBV in phylogroup III (*14*), and to determine the antigenic diversity of the glycoprotein (*15*). Given that IKOV is highly distinct from RABV (more genetically distinct than WCBV from RABV) and that a human has been bitten by an infected animal, the effectiveness of current rabies vaccines needs to be further investigated.
